# A Snapshot of an Innovative Approach towards Integrated Medical Biochemistry Teaching across the Pre-clerkship Curriculum for Impactful Outcomes

**DOI:** 10.12688/mep.21508.1

**Published:** 2026-04-21

**Authors:** Anamika Sengupta, Bei Zhang

**Affiliations:** 1 Health Sciences Education and Pathology (HSEP), University of Illinois College of Medicine at Peoria, 1.Illini Drive, Peoria, Illinois, 61605, USA; 2 Pathology and Laboratory Medicine, University of Vermont Larner College of Medicine, Burlington, Vermont, USA

**Keywords:** Medical Biochemistry, Pre-clerkship, Curriculum, Integration, Spiral, Longitudinal, Horizontal, Metabolism

## Abstract

Abstract: Medical biochemistry (MB) is usually taught at the inception of a medical curriculum with little to no clinical context. The timing of delivery and the antiquated teaching methods have portrayed MB in a negative light, limiting the appreciation of its importance in medical practice. To address the issue, the authors have developed a spirally integrated approach to teaching the subject, in which foundational MB topics are intentionally woven throughout the pre-clerkship curriculum, allowing students to revisit and deepen their understanding of the material in progressively more clinical and interdisciplinary contexts.

The above-mentioned approach included careful scrutiny of the curriculum to identify key interdisciplinary connections between selected biochemistry topics and other foundational and clinical sciences. This was followed by the development of clinical scenarios and a structured map for delivering the selected topics in a spiral fashion across multiple courses throughout the pre-clerkship curriculum.

The novel teaching approach aims to generate greater enthusiasm for MB among medical students, leading to a stronger appreciation of its role in medicine. The engagement of medical students with the material and the purposefully designed repeated encounter is envisioned to translate into noticeable improvement in students’ critical thinking and problem-solving abilities. The spiral approach to content delivery is designed not only to enhance content mastery but also to support broader cognitive skill development in medical students. Faculty members may also benefit from improved teaching effectiveness and greater appreciation from students.

This article provides a snapshot of this innovative teaching method, along with important tips for integrated instruction of a few common MB topics in the pre-clerkship phase of medical education. It also emphasizes the need to craft, implement, and evaluate a fully integrated MB curriculum blueprint as a resource for faculty development for medical educators worldwide.

## Introduction

### The educational challenge

Teaching and learning experiences in medical biochemistry (MB), one of the primary basic science subjects taught in the early pre-clerkship curriculum in U.S. medical schools, pose equal challenges for both medical students and educators. From the student’s perspective, the subject is cognitively demanding due to its voluminous content, long, winding pathways, and multifaceted metabolic cycles. Thus, unable to purposefully comprehend or appreciate its relevance to future medical practice, newly admitted medical students develop a negative bias towards the subject. As a solution, students habitually commit to rote memorization of the associated concepts to pass internal assessments and medical board exams. Consequently, by the time MB’s relevance approaches clarity during clinical years, self-recapping/relearning becomes an additional chore. This reinforces the negative bias towards the subject and prompts students to rely on “Google searches” for answers on demand.

From the educator’s perspective, teaching MB in a case-based or problem-based context to newly enrolled medical students with minimal medical knowledge presents challenges. Since most traditional curricula provide limited scope for reintroducing previously covered concepts into later discourse, the clinical application of MB concepts is almost always absent in a traditional pre-clerkship medical curriculum.

Harden ^1^ defines integration as “the organization of teaching matter to interrelate or unify subjects frequently taught in separate academic courses or departments”. When this organization spans across time and maintains continuity, it represents a “longitudinal” integration approach. When the organization occurs both within (intra) and amongst (inter) different disciplines within a curriculum, it represents “horizontal” integration. ^2^ Such integrative approaches can best be achieved in a “spiral” curriculum ^3^ characterized by spaced repetition of clinically relevant concepts. Each revisit, termed a “spiral”, aims to further elaborate on the original concept through more advanced intra-and/or interdisciplinary iterations.

Aiming to deliver MB with emphasis on the clinical relevance and inter/intra subject connections, while exposing students to the complexity of the subject at a gradual pace, we are invested in developing a detailed blueprint of an MB curriculum featuring horizontal and longitudinal integration, combined with a spiral delivery spanning across the pre-clerkship curriculum. We intend to share a brief segment of our innovative pilot teaching effort on a few common basic medical biochemistry topics with the Medical Education community worldwide through this article.

**TIPS FOR INTEGRATED TEACHING OF A FEW IMPORTANT MEDICAL BIOCHEMISTRY TOPICS**

1. **Teaching “Oxidative Fuel Metabolism” with an integrated approach**

*Initial presentation of Oxidative fuel metabolism (“X”)*: X includes core MB topics, namely glycolysis, the tricarboxylic acid (TCA) cycle, the electron transport chain (ETC), glycogenolysis, the pentose phosphate pathway, and their regulatory mechanisms, all of which are high-yield in the pre-clerkship curriculum. Traditionally, X is introduced to students at the inception of the curriculum with little to no clinical context. At this time point, students often fail to comprehend the practical or clinical relevance of “X” related concepts.

After the initial presentation of X, we made a conscious effort to integrate its associated subtopics throughout the pre-clerkship curriculum. This involved scrutinizing curricular materials through a multidisciplinary lens to identify key intra- and inter-disciplinary connections to X and associated clinical correlations (  Table 1). We invested time in developing clinical scenarios for each connection. We then carefully mapped the spiral delivery of X-related topics across different points in the curriculum (outlined below). Our teaching efforts aimed to convey to students the important message: “every pathological condition is caused by an altered physiology, which in turn is manifested by an inherited or induced biochemical/genetic alteration at the molecular level.”

**Table 1.** Ideas of spirally integrating “X” (oxidative fuel metabolism) with multiple disciplines across different time points of the pre-clerkship curriculum, post its presentation at the inception.

Initial delivery of “X” (Oxidative Fuel Metabolism): Inception of pre-clerkship curriculum; little to no clinical context				
Spiral delivery	Longitudinal integration in different Units/blocks	Horizontal integration with other disciplines	Identified topics for integration	Context of clinical integration
*Spiral 1*	Muscle and movement	Physiology	Skeletal muscle energetics	Marathon, Sprinting
*Spiral 2*	Cellular pathogenesis	Physiology, hematology, pathology	RBC metabolism, oxygen transfer, cancer cell metabolism	Hemolytic anemias, Warburg effect
*Spiral 3*	Cardiovascular and Renal	Physiology, pathophysiology	Cardiac muscle metabolism, renal tubular transport	Myocardial infraction, acute kidney injury
*Spiral 4*	Neurology	Cell physiology, neurobiology	Neurotransmitter biosynthesis	Multiple sclerosis

*Spiral-1: Integrating X with muscle energetics and muscle movement (physiology)*

Spiraling back concepts of glycolysis, TCA, ETC, and glycogenolysis from the introductory block of the pre-clerkship curriculum, into advanced discussions of energy metabolism of type I (slow oxidative) and type II (fast glycolytic) muscle fibers, highlighted the application of X in muscle energetics while showcasing horizontal integration between biochemistry, physiology and histology. Delivery of the content in the context of physical activities namely marathon (type I dominated) and sprinting (type II dominated), highlighted the role of X in athlete’s strength training, while integrating hormonal (insulin, glucagon, epinephrine) and enzymatic (via AMP kinase) regulation of muscle energetics and clinical treatment of the athlete’s dehydration (dextrose infusions).

*Spiral-2: Revisiting X in the context of hematology, cellular pathogenesis, and nutrition*

Integrating discussions of RBC metabolism, namely anaerobic glycolysis and the pentose phosphate pathway (PPP), into hematology sessions focused on hemolytic anemias allowed a review of X-linked concepts while applying them to the pathogenesis of hemolytic anemias caused by glucose-6-phosphate dehydrogenase (G-6-PD) deficiency and pyruvate kinase deficiency. The 2,3-bisphosphoglycerate (2,3-BPG) shunt associated with glycolysis was “spiraled back” into discussions of oxygen delivery by hemoglobin, the Bohr and Haldane effects (physiology), and the clinical contexts of thalassemia, sickle cell anemia, and hereditary persistence of fetal hemoglobin (HPFH). Revisiting glycolysis and TCA in the context of vitamin deficiency diseases like Beriberi, emphasizing the role of Vit B1 as a coenzyme for important mitochondrial enzymes such as the pyruvate dehydrogenase complex and alpha-ketoglutarate dehydrogenase, is another example of spiral integration. Glycolysis and PPP were again integrated into discussions of cancer pathogenesis in the context of the Warburg effect (altered metabolism of cancer cells), demonstrating a much higher level of horizontal integration among biochemistry, hematology, and pathophysiology.

*Spiral- 3: Integrating X with cardiovascular & renal physiology & pathophysiology*

A comparison of fuel choices between normal and ischemic cardiomyocytes in the context of myocardial infarction re-emphasized the role of cellular fuels (namely fatty acids, glucose, and ketones) in cardiac muscle function. This comparison also highlighted horizontal integration between MB, cell biology, histology, physiology, and pathology. Furthermore, concepts related to X were seamlessly integrated into discussions of tubular transport and intra-renal oxygenation mechanisms to highlight the differential metabolic needs of renal cortical and medullary regions. Such integration also highlighted the role of impaired fuel metabolism, driven by mitochondrial dysfunction, in the pathogenesis of acute kidney injury (AKI).

*Spiral-4: Integration of X with Neurology*

The versatility of the glycolysis and the TCA cycle in different human cell types were emphasized through focused discussions of excitatory and inhibitory neurotransmitter synthesis using glycolytic and TCA intermediates. Examples included glutamate synthesis from alpha-ketoglutarate, aspartate synthesis from oxaloacetate, gamma amino butyric acid (GABA) synthesis from glutamate and glycine synthesis using 3-phosphoglycerate. The discussions were framed within the context of multiple sclerosis.

2. **Teaching “glucose and lipid metabolism” with an integrated approach**

*Initial introduction of glucose and lipid metabolism in the context of “Important Metabolic States” of the body*

Fasting, fed, and starvation states are central metabolic conditions that anchor many high-yield concepts for medical board exams, including STEP-I and COMLEX-I in MD and osteopathic curricula. The fed state naturally frames discussions of glucose absorption, the distribution and kinetics of various GLUT transporters, the stimuli and mechanisms of insulin secretion from pancreatic β-cells, and the insulin-driven upregulation of anabolic pathways—glycolysis, glycogen synthesis, fatty acid synthesis, and protein synthesis—in hepatocytes, adipocytes, and muscle cells. These processes support energy production, nutrient storage, and overall metabolic homeostasis. When taught through the simple lens of what occurs after consuming a glucose-rich meal(fed state) enables rich, application-focused exploration of these pathways and encourages horizontal integration of medical biochemistry with physiology and introductory endocrinology.

**Figure 1.**
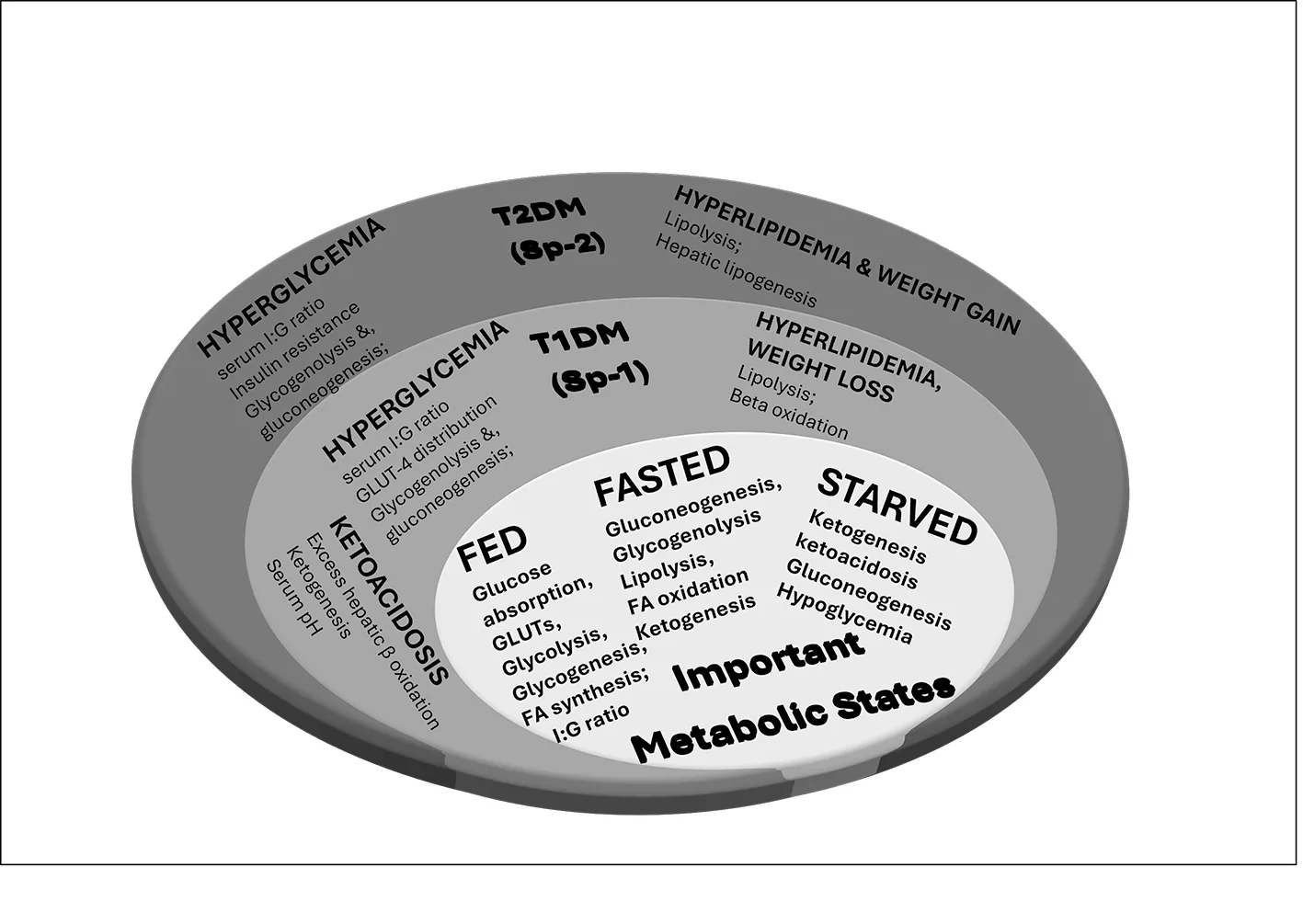
Spiral integration of glucose and lipid metabolism concepts with pathogenesis of Type 1 Diabetes mellitus (T1DM) and Type 2 Diabetes mellitus (T2DM). Abbreviations: I-Insulin; G-Glucagon; Sp-1: Spiral 1; Sp-2: Spiral 2

The fasted and starved states provide a clear framework for teaching catabolic pathways, including glycogenolysis, gluconeogenesis, lipolysis, fatty acid oxidation, and ketogenesis, as illustrated in Figure 1. Emphasizing the roles of glycogenolysis and gluconeogenesis in maintaining blood glucose homeostasis during fasting and early starvation, along with the hormonal regulation of these pathways by glucagon and epinephrine, supports high-yield, clinically grounded integration across biochemistry, cell biology, and basic endocrinology. Expanding on the increasing reliance on gluconeogenic precursors (lactate, pyruvate, alanine, aspartate), lipolysis, and fatty acid oxidation during prolonged starvation—and the progressive fuel shift from glucose to fatty acids and eventually to ketone bodies—highlights how metabolic biochemistry underpins the body’s adaptive survival strategies while conserving glucose for essential tissues.

*Spiral 1: Integrating glucose, lipid, and ketone metabolism to explain the pathogenesis of Type I Diabetes Mellitus (T1DM)*

Discussion of the pathogenesis of T1DM provides an effective framework for demonstrating the clinical relevance of core metabolic biochemistry concepts introduced at the start of the pre-clerkship curriculum. It links hyperglycemia (elevated blood glucose levels), hypoinsulinemia (driven by low insulin production or slow insulin secretion), and elevated glucagon levels to the resulting hypercatabolic state, characteristic of T1DM, driven by a cellular glucose deficit. This deficit activates multiple tissue-specific catabolic pathways, creating an opportunity to revisit adipose tissue lipolysis, fatty acid and ketone oxidation, hepatic ketogenesis, gluconeogenesis, and glycogenolysis as compensatory mechanisms induced in patients with T1DM, all aimed at mitigating the cellular energy crisis. These enriched spiral discussions support horizontal integration across biochemistry, endocrine physiology, and pathology in the context of T1DM pathophysiology, while also explaining clinical manifestations such as rapid weight loss and ketoacidosis ( Figure 1). They highlight how foundational metabolic principles connect directly to clinical applications within an integrated pre-clerkship curriculum.

*Spiral 2: Integrating glucose and lipid metabolism to explain the pathogenesis of Type 2 Diabetes Mellitus (T2DM)*

 The pathogenesis of T2DM is typically covered in the endocrine block near the end of the second year, when students have sufficient clinical background to understand the condition’s complications. This provides another opportunity to revisit insulin-driven anabolic pathways, such as fatty acid and cholesterol biosynthesis, in the context of hyperinsulinemia caused by insulin resistance. The hyperglycemia (high blood glucose level) caused by insulin resistance provides a framework for integrating concepts from cell biology and genetics, including mechanisms of receptor desensitization, inherited receptor defects, and abnormalities in downstream signaling molecules, into discussions of T2DM pathogenesis (  Figure 1). The associated hyperlipidemia further enables integration of adipose tissue endocrine functions, particularly leptin and adiponectin secretion, with insulin resistance. These discussions model broad, in-depth horizontal integration across biochemistry, cell biology, physiology, and endocrinology, while linking metabolic biochemistry concepts to their clinical relevance. The clear mechanistic connections between molecular pathways and real clinical diseases often energize students by showing how foundational knowledge directly explains patient presentations. Repeated exposure to these themes across the pre-clerkship curriculum also reinforces longitudinal curricular integration.

## Potential outcomes of our novel teaching initiative

1. **Potential benefits for medical students**
  • *Promotion of deeper understanding and longer-term retention of subject knowledge*
  Our novel integrated teaching effort of MB related concepts would help students correlate the conceptual knowledge (‘what’) with the strategic knowledge (‘how’) and the conditional knowledge (‘why’), thereby promoting deeper understanding, long-term memory, and better preparedness for medical board exams (USMLE STEP I and COMLEX-I) and their future clerkship education. As biochemistry educators, we strongly believe that a deeper, integrated understanding of metabolism doesn’t just help students pass exams in the medical curriculum, it helps develop a mechanistic mindset in medical students, which they carry into clinical practice. It also meaningfully shapes the kind of diabetes care they will deliver as future physicians.
  Such a model can also be easily adopted by other fundamental sciences.
  • *Fostering the development of critical thinking and problem-solving skills*
  The existing literature emphasizes the significant association among elements of the curriculum (content, objectives, methods, and assessments), factual knowledge, and a disposition toward critical thinking. ^4^ The pre-clerkship curriculum period is the only time in the entire professional career of an aspiring physician when the fundamentals of biomedical sciences and clinical skills (such as history-taking and physical exams) intersect and are formally taught and learnt. Through spaced repetition (spiral delivery), continual practice, and critical appraisal of interdisciplinary connections in clinical contexts, this curricular model would facilitate an earlier and more cohesive development of critical thinking and problem-solving skills in medical students, as was evident at our institutions (through their in-class interactions and internal exam performances). These crucial traits are expected to contribute significantly towards students’ success in clerkship education and future medical practice. ^5^
2. **Potential benefits for medical biochemistry educators**
  Indeed, we predict that participation in this model of teaching across multidisciplinary domains would provide a satisfactory teaching experience for biochemistry educators and would likely lead to greater recognition and appreciation from both medical students and medical school administrators. The model also has the potential to be easily adopted by other fundamental sciences and yield similar promising outcomes.
3. **Assembling an interdisciplinary curriculum team invested in a common goal**
  The integrated approach to teaching MB would facilitate the breakdown of interdisciplinary and inter-departmental boundaries, helping establish a cohesive team of faculty members and administrators all dedicated to a common goal of highly skilled classroom teaching.

## Conclusion: Aiming for a bigger gain in the future

Positive student feedback (as part of the regular student evaluation for these sessions) on the pilot integrated teaching effort for selected MB concepts has motivated the authors to develop a full roadmap for an integrated pre-clerkship MB curriculum. The planned curriculum will use updated materials, consistent delivery methods, and ongoing evaluation of its impact on student success. Once completed and published, the project will serve as a faculty development resource and help modern medical educators of the 21 ^st^ century move beyond the outdated two-pillar Flexner ^6^ model, which for years separated the delivery of foundational and clinical sciences in medical education.

## Ethical statement

This article reflects a snapshot of the authors’ educational initiative as medical educators and does not involve a research study. No human participants were recruited, no identifiable learner or patient data were collected, and no interventions were conducted for research purposes. Ethical approval was therefore not required. All reflections and examples are drawn from the authors’ professional experience.

## Data Availability

No data are associated with this article.
