# Supplementary material for: A Snapshot of an Innovative Approach towards Integrated Medical Biochemistry Teaching across the Pre-clerkship Curriculum for Impactful Outcomes
Source: MedEdPublish (2016). 2026 Apr 21;16:11. [Version 1] doi: 10.12688/mep.21508.1 (PMC13398181; doi:10.12688/mep.21508.1)
Supplement: Peer review history

# Reviewer report: Reviewer response for version 1

**DOI:** 10.21956/mep.23042.r45957

**Published:** 2026-06-17

**Authors:** Nasibah Azme

**Affiliations:** 1 Universiti Teknologi MARA (UiTM), Sungai Buloh, Malaysia

1. This is a well-written and informative educational innovation paper that provides valuable insight into integrating medical biochemistry into a pre-clerkship curriculum. The manuscript is well structured and the examples clearly explain the principles of spiral integration. 

 2. The difference between horizontal integration, longitudinal integration, and the spiral curriculum are clearly described, making a potentially complex educational framework easy to understand for readers from varying backgrounds. The authors make it clear that fundamental concepts in biochemistry are taught and learned again in increasingly sophisticated and clinically relevant applications, which is consistent with the philosophy of spiral curriculum.

 3. The following are some recommendations for improvement:

 - The authors can further strengthen the manuscript by discussing other literature on educational benefits of spiral curricula, including better knowledge retention, better integration of basic and clinical sciences, reinforcement of long-term learning, and better development of clinical reasoning and critical thinking skills. This would provide a stronger theoretical basis for the proposed educational approach. 

 - It may be helpful to explain how assessment strategies were designed in relation to the instructional practices of longitudinal and spiral integration. Any information on formative or summative assessments to reinforce integrated learning would strengthen the manuscript. The discussion on this can be considered for future implementation. 

If evidence from practice is presented, are all the underlying source data available to ensure full reproducibility?

Yes

Are all factual statements correct and adequately supported by citations?

Partly

Are the conclusions drawn balanced and justified on the basis of the presented arguments?

Yes

Are arguments sufficiently supported by evidence from the published literature and/or the authors’ practice?

Yes

Is the topic of the applied insights discussed accurately in the context of the current literature?

Yes

Reviewer Expertise:

Medical Education

I confirm that I have read this submission and believe that I have an appropriate level of expertise to confirm that it is of an acceptable scientific standard.

# Reviewer report: Reviewer response for version 1

**DOI:** 10.21956/mep.23042.r45832

**Published:** 2026-06-09

**Authors:** Krystal Hunter

**Affiliations:** 1 Cooper University Hospital, Camden, New Jersey, USA

Summary of paper:

 Traditional medical schools teach medical biochemistry (MB) as a foundational course in a students first year of medical school without a clinical context.  This fact has lead to a negative perception of the course and limits the appreciation of the relevance of this course in a clinical context.

 The authors of the paper are presenting a novel way to reshape the MB curriculum so that there can be a foundational course that is expanded to clinical context where MB concepts can be applied to clinical contexts.  This will be beneficial to students and faculties. 

 The authors stated that they were “ *Aiming to deliver MB with emphasis on the clinical relevance and inter/intra subject connections, while exposing students to the complexity of the subject at a gradual pace, we are invested in developing a detailed blueprint of an MB curriculum featuring horizontal and longitudinal integration, combined with a spiral delivery spanning across the pre-clerkship curriculum*.”

 The areas where they used this in the following contexts:

 Teaching “Oxidative Fuel Metabolism” with an integrated approach

 Teaching “glucose and lipid metabolism” with an integrated approach

 Feedback:

 Strengths:

- The authors provided a detailed template of what the new course would consist of.
- Also, the authors used concepts that have been used in education in general and in medical education.  Particularly the use of steady repetition which was confirmed by Zhan et al where it was found that *when stimuli are learned by repetition, they are remembered better and retained for a longer time*.   
- The methodology by which the authors used to implement the expanded course is sound.  The expectations that they stated within the paper has been what has been reported in the education literature. 
- Used student feedback to refine the curriculum and is going to continue refining the course based on future student feedback. 
- Plans on using for training materials for faculty/facilitators (Like looking into the future to be sure that the course will still be effective.

Some areas of improvement:

- Would like to see the team also get feedback from faculty
- Would like to see more references to literature within the paper (although it is clear that educational theories were used in the formulation of the pilot program)

In conclusion, the work was very good.  Structurally, it was very easy to follow and made it easy to follow.  Also, this is a novel process that is based on previous evidence.  The only things that need improvement are very minor.

If evidence from practice is presented, are all the underlying source data available to ensure full reproducibility?

Partly

Are all factual statements correct and adequately supported by citations?

Yes

Are the conclusions drawn balanced and justified on the basis of the presented arguments?

Yes

Are arguments sufficiently supported by evidence from the published literature and/or the authors’ practice?

Yes

Is the topic of the applied insights discussed accurately in the context of the current literature?

Yes

Reviewer Expertise:

Biostatistics

I confirm that I have read this submission and believe that I have an appropriate level of expertise to confirm that it is of an acceptable scientific standard.
